# Habitat and indigenous gut microbes contribute to the plasticity of gut microbiome in oriental river prawn during rapid environmental change

**DOI:** 10.1371/journal.pone.0181427

**Published:** 2017-07-17

**Authors:** Cheng-Yu Chen, Po-Cheng Chen, Francis Cheng-Hsuan Weng, Grace Tzun-Wen Shaw, Daryi Wang

**Affiliations:** 1 Biodiversity Research Center, Academia Sinica, Taipei, Taiwan; 2 Institute of Fisheries Science, College of Life Science, National Taiwan University, Taipei, Taiwan; 3 Department of Life Science, National Taiwan Normal University, Taipei, Taiwan; International Nutrition Inc, UNITED STATES

## Abstract

Growing evidence points out that the capacity of organisms to acclimate or adapt to new habitat conditions basically depends on their phenomic plasticity attributes, of which their gut commensal microbiota might be an essential impact factor. Especially in aquatic organisms, which are in direct and continual contact with the aquatic environment, the complex and dynamic microbiota have significant effects on health and development. However, an understanding of the relative contribution of internal sorting (host genetic) and colonization (environmental) processes is still unclear. To understand how microbial communities differ in response to rapid environmental change, we surveyed and studied the environmental and gut microbiota of native and habitat-exchanged shrimp (*Macrobrachium nipponense*) using 16S rRNA amplicon sequencing on the Illumina MiSeq platform. Corresponding with microbial diversity of their living water areas, the divergence in gut microbes of lake-to-river shrimp (CK) increased, while that of river-to-lake shrimp (KC) decreased. Importantly, among the candidate environment specific gut microbes in habitat-exchanged shrimp, over half of reads were associated with the indigenous bacteria in native shrimp gut, yet more candidates presented in CK may reflect the complexity of new environment. Our results suggest that shrimp gut microbiota has high plasticity when its host faces environmental changes, even over short timescales. Further, the changes in external environment might influence the gut microbiome not just by providing environment-associated microbes directly, but also by interfering with the composition of indigenous gut bacteria indirectly.

## Introduction

Animals live in intimate association with diverse communities of symbiotic microorganisms [1, 2]. The intestinal microbial communities are particularly abundant and diverse, and the microbes contribute several important functions to their hosts, such as promoting host development, nutrition, and immunity [3–8]. Establishing and maintaining beneficial interactions between the host and its associated microbiota are key requirements for host health. Furthermore, the sum of the genetic information of host and its microbiota is defined as the “hologenome”, and the hologenome theory suggests that the adaptation and evolution of higher organisms cannot be well described without considering their microbial symbionts [9]. Growing evidences have pointed out a role of the gut microbiota acting as a key driver of hosts’ phenotypes, and the complete set of phenotypes (including host and microbes) is referred as phenomes [10]. A hypothesis developed from that perspective suggests that the capacity of the gut microbial composition or gene-expression pattern may change in response to the host's physiological changes, thus contributing to the phenomic plasticity [10, 11]. These aspects imply that variation of the external environment is likely an essential factor, which facilitates host acclimation and adaptation to environmental change through the change of gut microbiota. Therefore, to understand the hologenome modification in response to environmental change, it is crucial to investigate the factors associated with the changes of microbial community, especially in a rapidly changing environment.

As aquatic organisms are in direct and continual contact with the aquatic environment, the complex and dynamic populations of microbiota have particularly significant effects on host health and development [6], and are involved with host physiology, ecology, and even evolution [12, 13]. Increasing demand for aquaculture products has prompted the investigation of bacterial composition in the intestinal tract of key aquatic organisms. Aquaculture research has shown that microbes in the intestines of aquatic organisms contribute to the development of host immune and digestive systems [14–16]. Recently, investigations using Next-Generation Sequencing on 16s rRNA have shown that host genetic divergence strongly shapes the composition of gut microbiome [6, 7, 17, 18], meanwhile, distinct environments and diets also cause significant impacts on gut microbes [19]. This new technology has paved the path to exploration of the deeper relationship between hosts and gut symbiotic microbes. A recent study on gut microbiota of freshwater shrimp showed that the host genetics was a main contributor to the divergence of shrimp gut microbiomes between two closely related species, while the host habitat type seemed to play a critical role when comparing the microbial variation of hosts belonging to different lineages [20]. On the other hand, a survey of stickleback fish indicated that population effects were greater than habitat effects in explaining gut microbial variation [21]. This among-population difference was associated with multiple covarying ecological variables, including habitat type, lake geomorphology, and food-associated microbes, and it seems to depend more on internal sorting processes related to host genetics than on transient environmental effects such as colonization. These inconsistent results suggest that the effects on gut microbiota could be species specific, and further investigations will be required to test the relative contributions of internal host sorting and external colonization processes on gut microbiota.

*Macrobrachium nipponense*, a non-obligatory amphidromous prawn [22], originated in mainland China [23] and is broadly distributed over East Asian (China, Japan, Korea, Vietnam, Myanmar, and Taiwan) [24–26]. This oriental river prawn, which reproduces easily and is highly tolerant to various environments [25], has the potential for great economic value as a farmed shrimp [27]. Field research describes a group of oriental river prawns dwelling in the rivers to complete their life cycle, whereas other populations are found in inland freshwater lakes, and this ecological difference is known to cause physiological effects on the shrimp (*M*. *nipponense*), such as a smaller female size in the river population than in the lake population [25, 28, 29]. The observations suggest that this freshwater shrimp exhibits different phenotypes depending on environmental conditions [20], and therefore provides a feasible model to study whether the divergence in gut microbiome behaves as a driving force of host phenomic plasticity in shrimp. Our previous survey indicated that many gut symbionts presented environment specific profiles. For instance, by comparing the gut microbial composition of shrimps collected from rivers and lakes, the number of shared OTUs was only 299 (about 47 and 65% among lake and river populations, respectively), and the bacterial families *Vibrionaceae* and *Flavobacteriaceae* appeared to be responsible for the separation of lake and river groups [20]. In order to trigger changes in the shrimp gut microbes in response to rapid environment change, we performed a habitat exchange experiment. We aimed to determine whether the microbial diversity in gut, which potentially reflects the host phenomic plasticity, varies in response to the environmental type. Variation of water or sediment microbial diversity between the habitats was expected as one of the major differences in environment. Secondly, we proposed to determine the origin of the gut microbes. Microbial colonization of the animal gut begins at birth and continues throughout life; microbes can be obtained from parental inheritance, from ingested materials including water and diet, or via contact with other environmental factors, such as river sediment [21, 30–32]. Since the environmental specific bacteria may be key elements in shaping host phenotypes during acclimation and even adaptation to new environmental conditions, it is crucial to identify their origin. Our results indicate a strong correlation between gut microbiota and environmental complexity. We found that when shrimp were exposed to a new habitat with higher microbial biodiversity, both environmental and indigenous gut microbes contribute to the plasticity of shrimp gut microbiome, consequently leading to higher composition variation and divergence among individuals; yet when shrimp were transplanted to an environment with lower microbial diversity, only indigenous gut microbiota function as a major contributor, leading to a convergence of between-host (within-group) diversity. These findings give us the opportunity to infer the potential benefits of receiving microbes from water with higher diverse microbiota.

## Results

### Gut microbial divergence among shrimp groups

To study the role of the gut microbiota in habitat acclimatization, we chose *Macrobrachium nipponense* as a model species to investigate (i) whether gut microbial communities changed after habitat-exchange (between river and lake for one month), and (ii) whether habitat-exchanged shrimp received new colonization from environmental microbiota. By applying Miseq amplicon sequencing, the microbiomes from the guts of four groups of oriental river prawn (two native shrimp groups, two habitat exchange groups) and two types of environmental samples (water and sediment) were collected from Chengcing Lake (C) and Kaoping River (K) for analysis. Using the Chao1 estimator, our results showed that there is no significant change in community richness after habitat-exchange manipulation for one month (e.g. six river shrimp K vs. five river-to-lake shrimp KC, *P* = 0.153; three lake shrimp C vs. five lake-to-river shrimp CK, *P* = 0.470; S1 Table), although the microbial compositions had changed. To compare the microbial diversity between groups (among-group variation), the Shannon diversity was calculated, the results also indicated no significant difference among shrimp groups (S1 Table). Nevertheless, we observed that the lake-to-river group (CK) tended to possess a higher gut microbial diversity comparing with that of the native lake group (C), with marginal significance (*P* = 0.087).

### Compositional change of gut microbiota after habitat exchange

**Fig 1:**
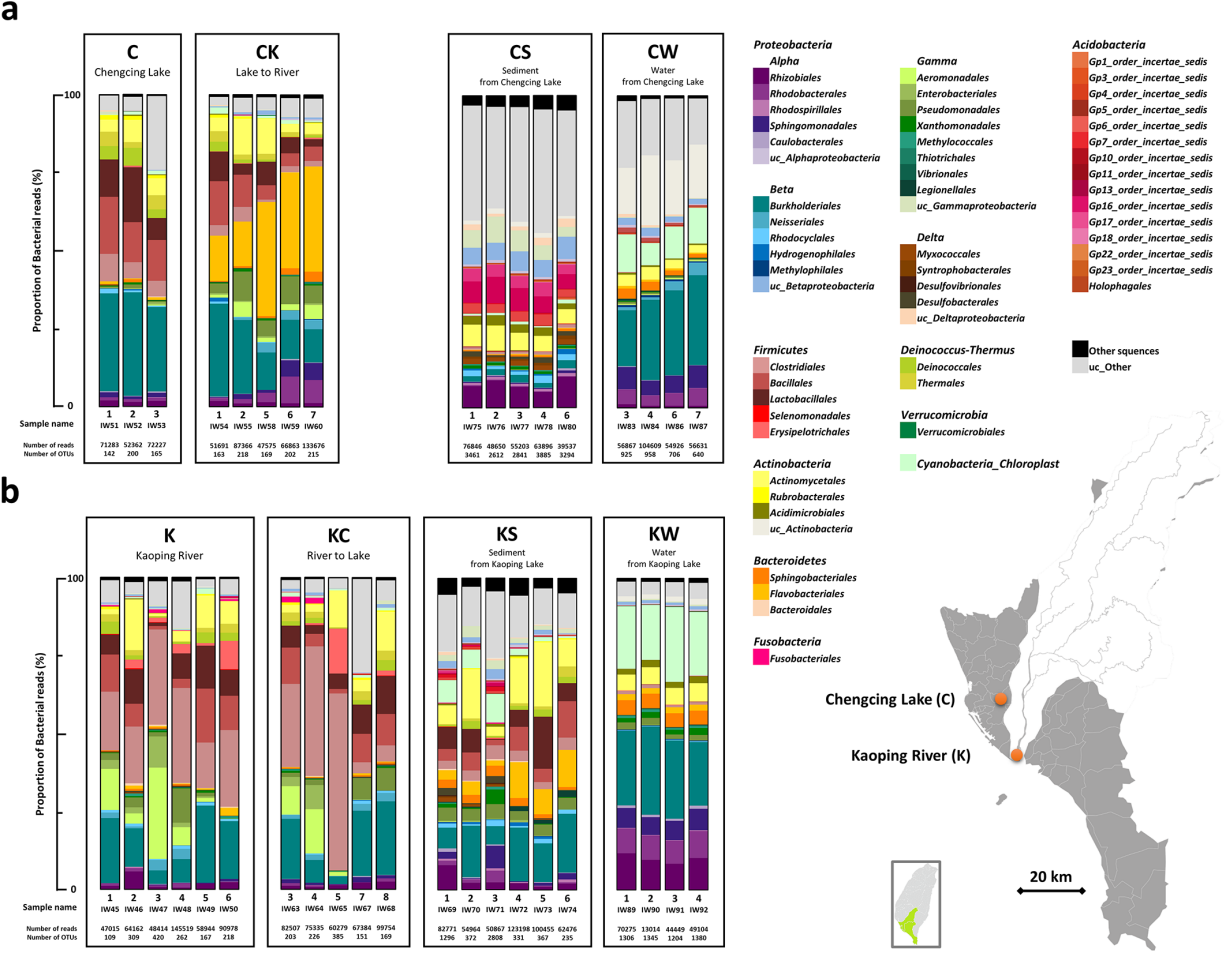
The microbial communities found in different gut samples of shrimp and environment samples. Number of reads, OTUs, and microbial composition of shrimp gut, water, and sediment from two types of habitat: Chengcing Lake (C) (**a**) and Kaoping River (K) (**b**). The percentage of sequences in each sample is classified to order by color, with phylum indicated at the top of each ordinal label. ‘un’: unclassified order.

Previous work has suggested that *Proteobacteria* is the major phylum in the gut microbiota of oriental river prawn *M*. *nipponense*, followed by *Firmicutes* and *Actinobacteria* [20]. Overall, our survey on gut microbial composition presented similar results (S1 Fig), while over 70% of reads were classified to phyla *Proteobacteria* and *Firmicutes* in 12/14 of K, KC, and C libraries, yet only 57.84% of total reads on average in the CK library, demonstrating striking changes of gut microbial composition in lake-to-river shrimp (CK) compared with the other three groups (Fig 1). Notably, the *Flavobacteriales* clade (*Bacteroidetes*; Fig 1, S1 Fig) became predominant (about 26.04% of reads) in the gut bacterial communities of CK shrimps, while representing less than 1% of reads in the native lake (C) library. The origin and the reason for the sudden expansion remain unclear.

**Fig 2:**
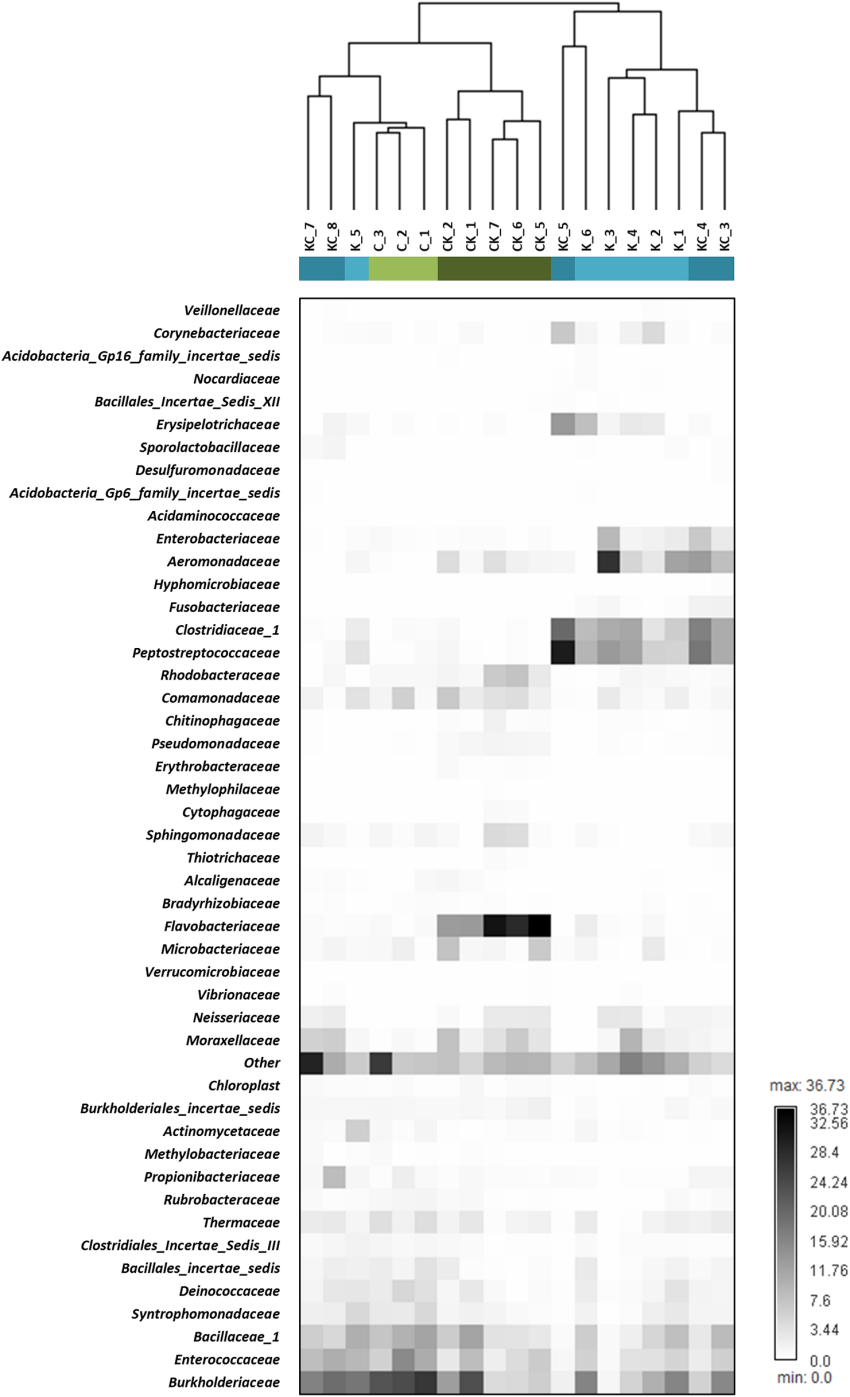
Frequency of OTUs in the shrimp gut microbiome represented as a heatmap. The OTUs were analyzed at the family level. Only families that made up more than 1% of the sequences of the libraries and had a greater than 2 fold change after habitat-exchange manipulation are represented. The hierarchical clustering tree above the data matrix was generated by average-linkage analysis of nineteen samples representing four shrimp groups.

To determine the impact of changed habitat on the shrimp gut microbiome, we performed hierarchical clustering using relative abundance data of OTUs at the family level. The results showed that when shrimp experienced habitat exchange, the gut microbial compositions still presented a higher correlation with those of shrimp from the original habitat. For example, as shown in Fig 2, the habitat-exchanged individuals (lake-to-river, CK_1, CK_2, and CK_5–7) have gut microbial compositions closer to that of native river shrimp (C_1–3) in the hierarchical clustering. Similarly in the other habitat-exchanged group, river-to-lake, individuals (KC_3–5) also clustered with native lake shrimps (K_1–4 and K_6), although there were some exception (e.g. river-to-lake, KC_7–8 clustered with native lake shrimps). Possible explanations are discussed later. Interestingly, although gut communities of lake-to-river shrimp (CK) were separated from that of native lake shrimp (C) (Fig 2), the three most dominant families (*Burkholderiaceae*, *Bacillaceae_1*, and *Enterococcaceae*) were the same. Correspondingly, *Peptostreptococcaceae* and *Clostridiaceae_1* were more prevalent among river and river-to-lake shrimps (K and KC).

**Fig 3:**
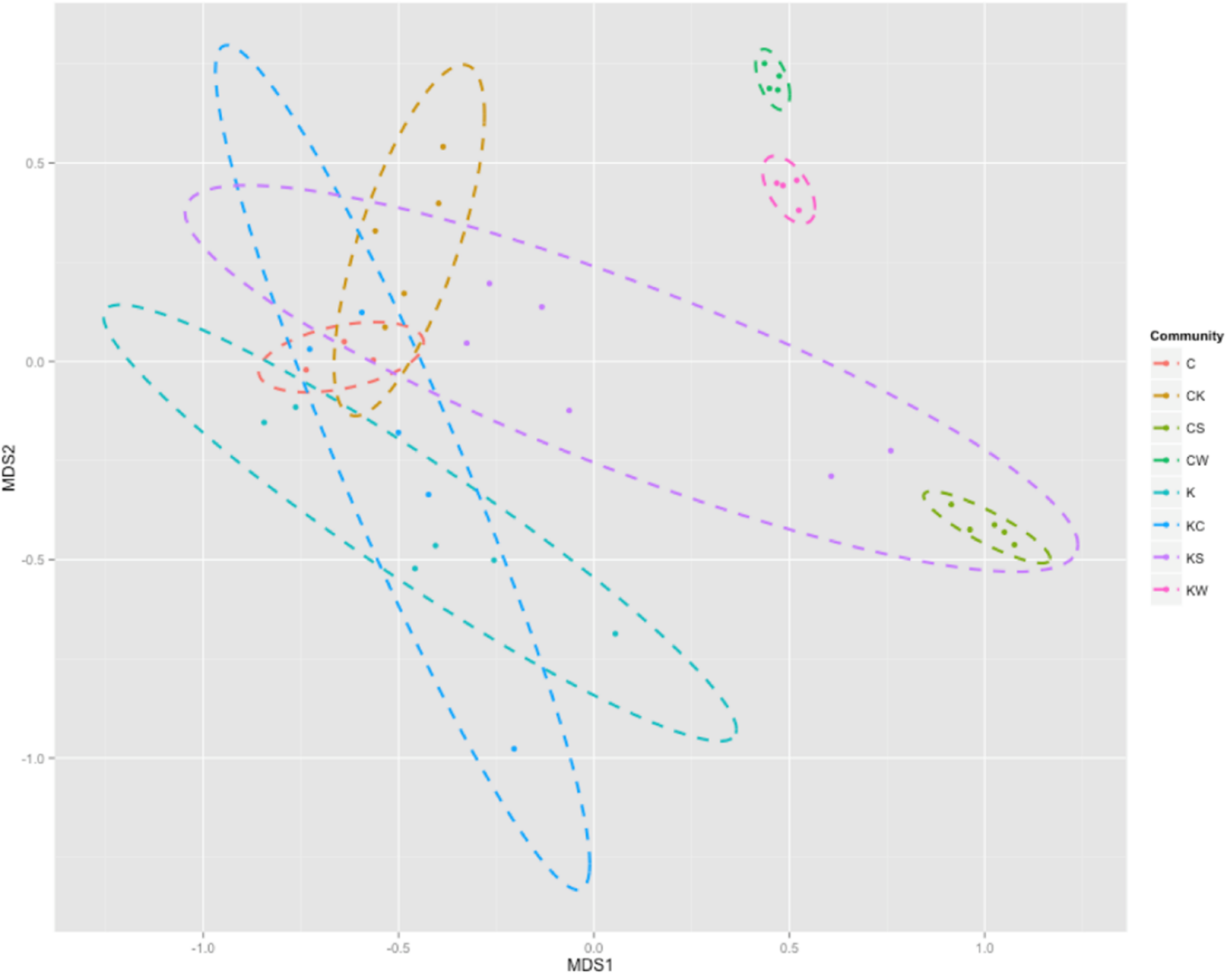
NMDS plot showing clustering among gut, water, and sediment samples based on OTU abundance classified at family level. Created with R package vegan, gplots, and RcolorBrewer.

Next, to better visualize the grouping and differentiation among bacterial communities of shrimp guts, water, and sediment across habitats, non-metric multidimensional scaling (NMDS) ordination at the family level was plotted for discussion (Fig 3). Most impressively, these gut bacterial surveys from across two habitats revealed modestly diverse gut communities among the shrimp (within-group variation) sampled from native habitat (C and K) compared with habitat-exchanged shrimp (CK and KC), and there were relatively independent between two native-habitat groups. Moreover, higher within-group variance was presented in the shrimp from Kaoping River (K and KC; seen as a wider span in the NMDS plot, Fig 3). These results can be explained by the fact that food resources are more complicated in rivers than in lakes [33], hence the results may reflect the importance of environment on complexity of gut microbiota of *M*. *nipponense*.

### More unique microbes in river shrimp

**Fig 4:**
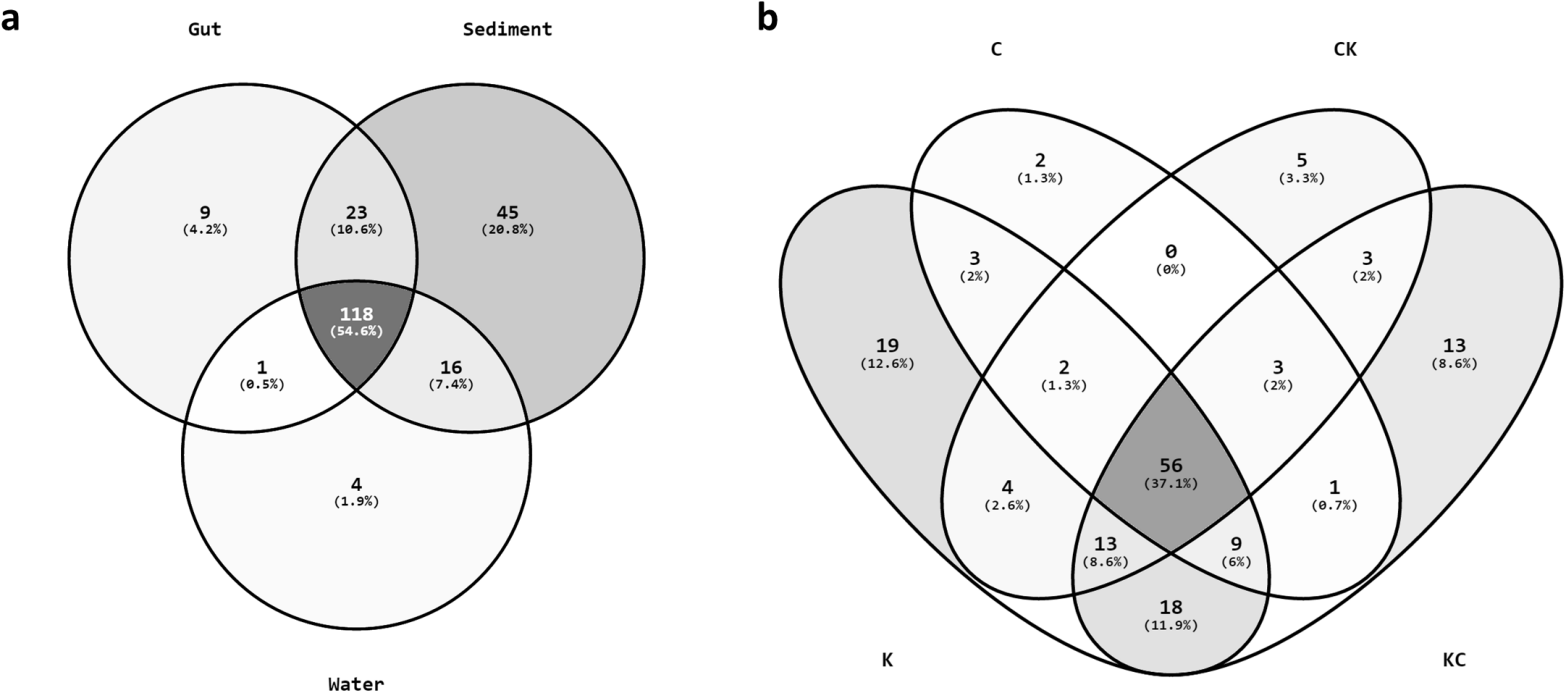
Shared OTUs. Numbers of shared OTUs classified at the family level among shrimp gut and the environment (**a**), as well as in four shrimp groups (**b**).

We next revealed the sharing of the microbial compositions in guts of two native shrimp (C and K), two habitat-exchanged shrimp (CK and KC), and environmental samples (CW, CS, KW and KS), in different combinations (Fig 4, S2B Fig). Considering all communities, only 3.43% of OTUs were shared among shrimp guts and the environments, and sediment samples contained the highest unique OTUs ratio (64.79%) (S2 Fig). A comparable proportion of gut microbial OTUs were shared with sediment (990/1666, 59.42%) and water (453/1666, 27.19%) microbial communities. Note that these counts of shared microbial OTUs might be sensitive to sampling bias, especially since the diversity of lake and river sediment may increase as sampling spots increase (Fig 3). While, at the family level, up to 92.38% and 78.81% of a total of 151 bacterial families from shrimp gut communities could be found in sediment and water, respectively (Fig 4A). Among these families, about 37% were overlapping in all shrimp groups (45.16% of K, 73.68% of C, 65.12% of CK, and 48.27% of KC; Fig 4B) and more unique families were present in the river shrimp (12.58% and 8.61% of the total in K and KC, respectively). These findings are consistent with previous results for microbial diversity in guts and environments (S1 Table, Fig 3) and provide evidence for a strong interplay between microbes in shrimp gut and the environment.

### Shrimp gut microbial community covaried with environmental microbiota

Oriental river prawn populations live in various environments such as lake, river, and estuary, and this ecological difference is known to cause physiological effects on *M*. *nipponense* [25, 29]. The divergent sources of microbial colonist might therefore alter the composition of shrimp gut microbes. The analysis of microbial community diversity indicated that environmental samples (water and sediment) from Kaoping River presented significantly higher variation compared with those of Chengcing Lake (*P* < 0.05) (S3 Fig), which seems to imply that the higher diversity of river-associated microbiota confers greater gut microbial variation in the lake-to-river shrimp population. Here, we emphasize that this result has low power, replying on only diversity estimates.

### Complex source of microbe contribution to the gut bacteria colonization in lake-to-river shrimp

**Fig 5:**
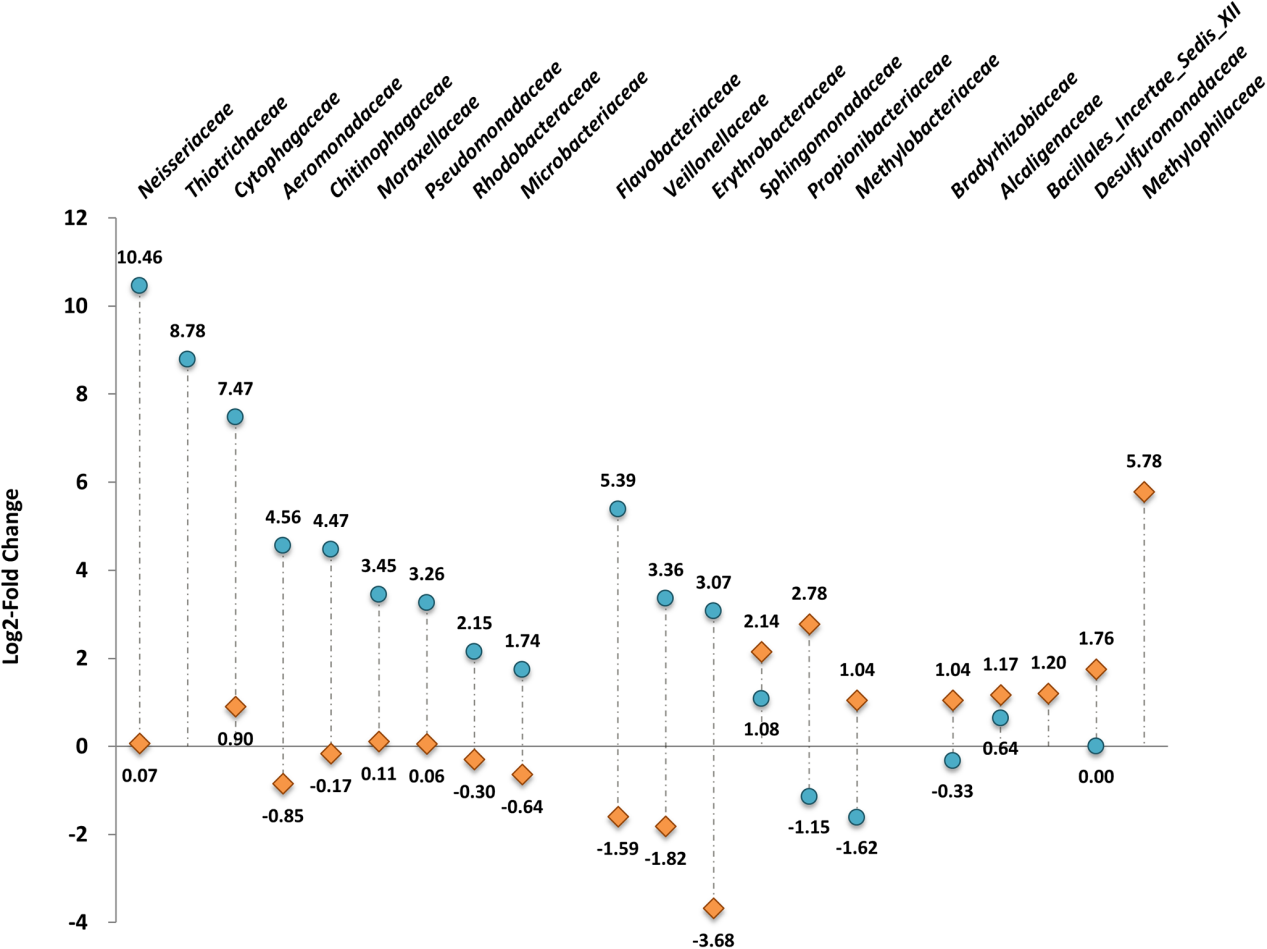
The frequency changes of crucial gut bacterial families in freshwater shrimp during habitat change. The log2 fold change estimate was used to quantify the difference of family-level OTU abundance in habitat-exchanged shrimp compared with the shrimp from native habitats, and candidates (targeted OTUs) with fold change ≥ 2.00 (|log2 ratio| ≥ 1) were regarded as biologically meaningful. The blue circle and orange rhombus correspond to the ratio CK/C and KC/K, respectively.

In order to evaluate the contributions of water- and sediment-associated microbes to the shrimp gut microbiome, we first focused on the OTUs (at the family level) that exhibited dramatic change after habitat exchange. The log2 fold change estimate was used to quantify the change of OTUs abundance. After the analysis, twenty targeted OTUs with fold change ≥ 2.00 (|log2 ratio| ≥ 1) were regarded as biologically meaningful and were selected for further analysis (Fig 5). As expected, the result from the fold change estimation was consistent with the above-mentioned composition analysis (Fig 4); the lake-to-river group (CK) contained more OTUs with fold change ≥ 2.00, compared to the river-to-lake group (KC). CK had 13 OTUs with 3.35- to 1406.56-fold abundance change, while KC had 8 OTUs with 2.06- and 54.96-fold change in river shrimp. It is worth noting that the average relative abundance of families *Flavobacteriaceae*, *Veillonellaceae*, and *Erythrobacteraceae* in the gut of lake-to-river group had increased by 41.91, 10.25, 8.40 times, yet decreased in river-to-lake shrimp by 3.01, 3.52, and 12.78 times; the families *Propionibacteriaceae* and *Methylobacteriaceae* had 6.85- and 2.06-fold increases in river-to-lake shrimps while were 2.21- and 3.06-fold decreased in lake-to-river shrimps, respectively. Especially, only the family *Sphingomonadaceae* became dominant in both habitat-exchanged shrimp (2.11-fold increase in lake-to-river and 4.12-fold increase in river-to-lake shrimp).

**Fig 6:**
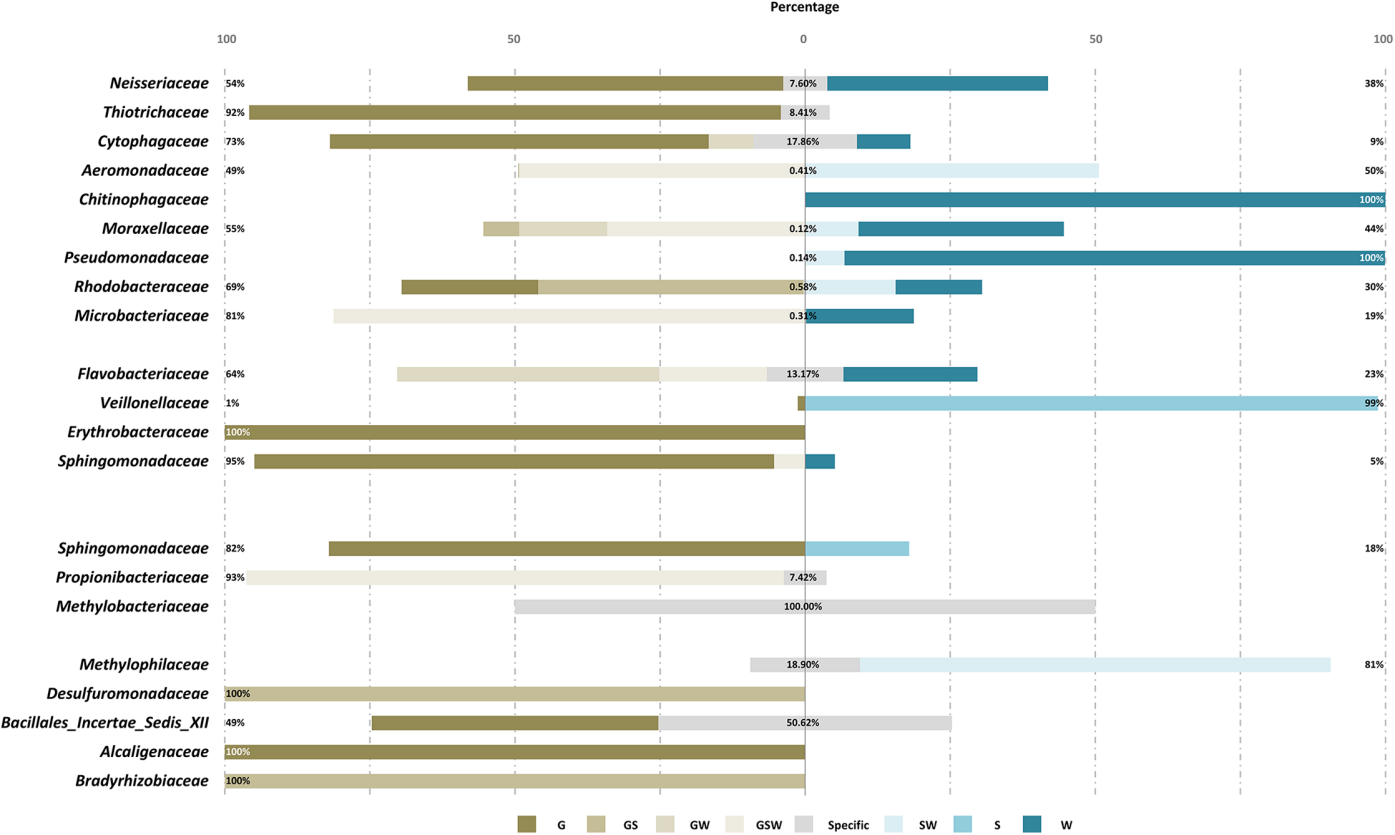
Percentage of reads shared between gut and environment microbial communities increased more than two folds after habitat-exchange manipulation. The source of the microbes represented by reads were divided into G (shared with the gut microbiota from native habitat), S (shared with sediment microbiota from the water area of habitat-exchange manipulation), W (shared with water microbiota from the water area of habitat-exchange manipulation), SW (shared with both sediment and water microbiota from the water area of the habitat-exchange manipulation, etc.), GS, GW, GSW, and Specific (unshared core OTUs of habitat-exchanged shrimp) groups.

**Fig 7:**
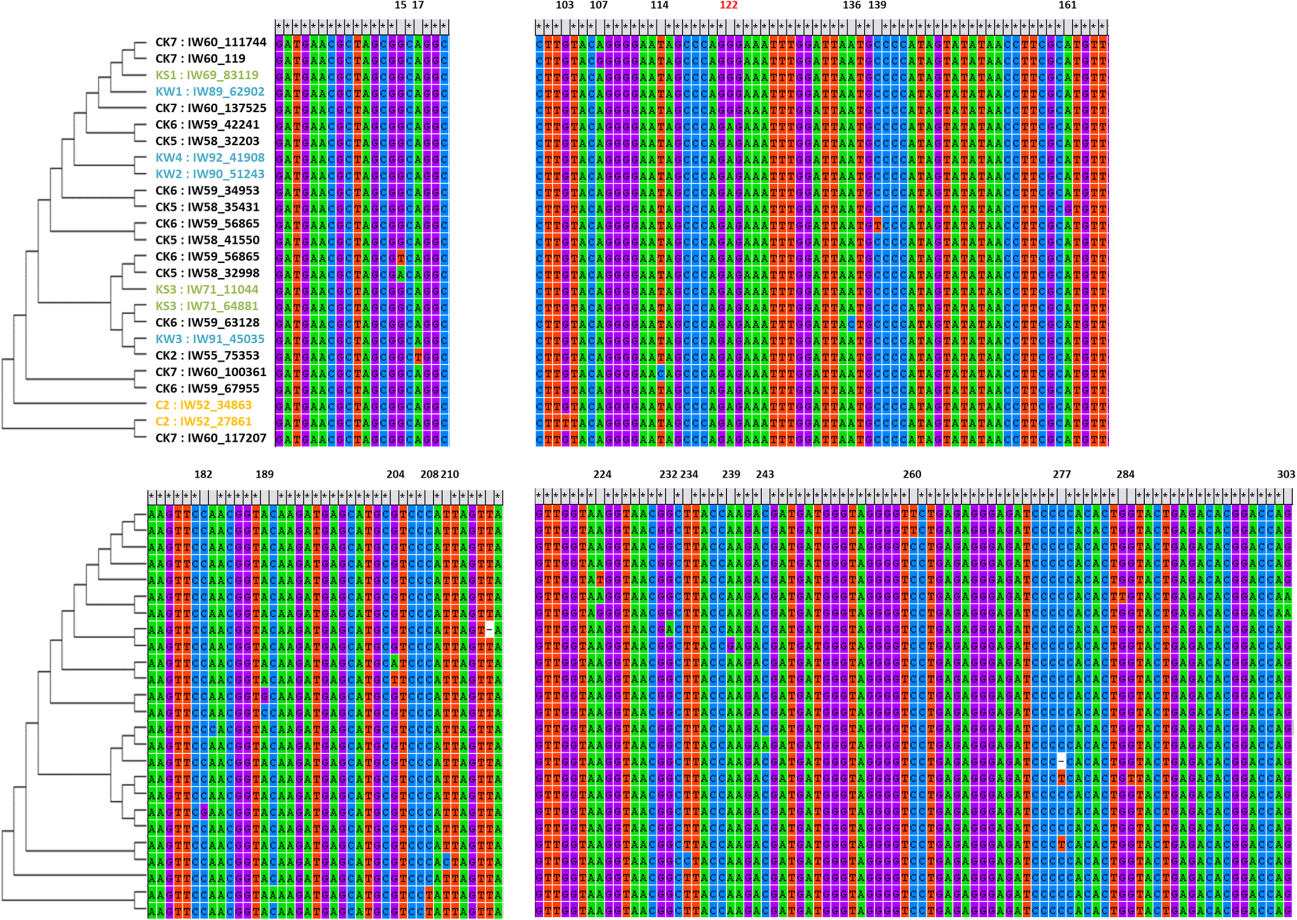
Alignment and phylogenetic tree of reads among OTUs with 99% similarity of family *Flavobacteriaceae* from gut and environmental samples. The reads of each sample with 99% similarity were randomly chosen and aligned for comparison. The tree was constructed using neighbor-joining method with 1000 bootstrap replicates in MEGA 6.06 program.

Variations in gut microbiota composition during habitat exchange might result from (i) an enrichment of rare population of native commensal gut microbiota or (ii) a colonization by environmental microbiota. To better understand the origins of gut bacteria in habitat-exchanged shrimp, a sequence-based analysis using USEARCH global alignment algorithm was performed. Our results indicated that after habitat exchange (CK and KC), three quarters of targeted OTUs (15/20) had over 50% of reads associated with the native shrimp gut (Fig 6), reflecting a native origin of those OTUs. This is a first observation demonstrating the importance of native commensal (indigenous) microbes in shrimp guts when shrimp move to a new environment where the same microbes are also available. It’s interesting to note that in lake-to-river shrimp (CK), the reads of some OTUs were more dominantly associated with environmental microbes, especially water-associated colonists, compared with those in river-to-lake shrimp (KC). For instance, over 90% of reads classified as families *Aeromonadaceae*, *Chitinophagaceae*, *Moracellaceae*, *Pseudomonadaceae*, or *Microbacteriaceae* in CK were shown to originate from water-associated microbes in Kaoping River, and 99% of reads classified as *Veillonellaceae* were associated with sediment microbes. Alternatively, in river-to-lake shrimp (KC), only two OTUs (*Sphingomonadaceae* and *Methylophilacaea*) were found to be associated with the environmental microbes of the new habitat. Furthermore, we noticed that the most dominant OTU (*Flavobacteriaceae*) of the CK gut community deserves more attention. In this case, the reads had multiple origins, including 23% associated with water microbes in the new habitat, 64% associated with the commensal gut microbiota of native lake shrimp (C) and also native water, and about 13% specific to this group. The phylogenetic analysis for reads we randomly chose from this OTU (*Flavobacteriaceae*) provided evidence to clarify the origin of gut microbes when experiencing environmental perturbation (Fig 7); the multiple sources might indicate that complex processes have been executed in shrimp gut during habitat exchange.

## Discussion

In recent years, studies on the interaction between hosts and their gut microbiota have drawn much attention in microbiome research. In humans, it is known that the diversity and richness of gut microbiota are established mainly in early childhood, and family members tend to harbor more similar microbiota, which could be due to a shared living environment and may also reflect host genetic relatedness [34]. In aquatic animals, host populations exposed to different microbial communities may exhibit divergent microbiota. For instance, fish gut microbiota differ between fresh and marine populations [31], and even between river and lake populations [21]. Moreover, an entirely different gut microbial composition has been shown in lab-reared fish compared with wild fish [18], providing more evidence of the effects of diet and environment on gut bacterial communities. Here, we focused on the compositional change of gut microbiota in shrimp facing rapid environmental change, and investigated the specific contributions of indigenous versus environmental microbes to the change of the gut microbiota. Our data demonstrate that river and lake shrimp differed in its symbiotic gut microbial composition and within-group (between-host) diversity. Although there was no significant difference in gut microbial diversity after habitat exchange, we found that in response to the complexity level of environmental microbiota (water, sediment of river and lake), the within-group diversity of gut microbes increased in lake-to-river shrimp, but decreased in river-to-lake shrimp. In habitat exchange, many symbiotic microbes were also changed during the acclimation process. A typical example is *Flavobacteriales*, which was a low abundance clade in lake shrimp but turned out to be the major clade in lake shrimp reared in the river environment for one month. Coincidently, the gut commensal *Flavobacteriaceae* was found to reside in all shrimp dwelling in different rivers [20], implying a critical role of this bacterial family in gut of shrimp living in river habitats. In addition, river (K), river-to-lake (KC), and even the lake-to-river (CK) shrimp had higher within-group variance of gut microbiota than the shrimp living in native lake habitat (C), reflecting a dramatic effect associated with the river habitat. These observations provided a first glimpse of how the bacterial community in shrimp guts was affected by rapid environmental change.

Environmental variations, such as dietary source, abundance of preferred diet, or environmental temperature, are known to cause fast and significant changes in gut microbiomes [18, 21, 35, 36]. Bayesian community-level source tracking, a computational approach, was developed to estimate the ratio of the gut microbes potentially obtained from water or prey source in their habitat [21, 37]. However, the contribution of environmental and indigenous gut microbes to gut microbiota in animals facing external environmental change has not been previously investigated. Our present study investigated the origin of shrimp gut microbes in habitat-exchanged individuals. Of gut microbes that showed proliferation in response to habitat exchange, a high proportion was associated with the indigenous gut bacteria in native shrimp. This suggests that external environmental change might influence the phenomic plasticity not just by harboring environment-associated microbes directly, but also by enriching rare species of indigenous gut bacteria, which might result from the alteration of host physiological conditions such as the gut nutritional environment [38, 39] and energy homeostasis [35]. Note that the habitats in this survey differ in abiotic variables including water chemistry and temperature (S2 Table), both of which may separately affect identify and population levels of environmental microbes, as well as physiology and then gut microbial colonization of the host facing a new habitat. Correspondingly, our inference is also supported by a recent hypothesis that suggests the gut microbial composition may change in response to variations of the host's physiology and external environment over short timescales, and more importantly, this capacity change is likely to be an essential factor in host acclimation and adaptation [10]. Furthermore, we found a higher percentage of newly acquired gut bacterium from the environment was observed in lake-to-river shrimp compared with that in river-to-lake shrimp. Our results also support a strong correlation between gut microbiota and environmental complexity. We inferred that, when shrimp were exposed to a new habitat with higher microbial biodiversity, both environmental and indigenous gut microbes contribute to the plasticity of shrimp gut microbiome, consequently, leading to higher composition variation and divergence among individuals; by contrast, when facing lower microbial diversity, only indigenous gut microbiota play as a major contributor, leading to a convergence of between-host diversity.

Interestingly, several potential pathogens of shrimp [40, 41], such as *Aeromonadaceae*, *Moraxellaceae*, *Pseudomonadaceae*, and *Flavobacteriaceae*, were among the targeted gut microbes (with fold change ≥ 2.00) associated with habitat exchange in lake-to-river shrimps. For example, the family *Pseudomonadaceae*, as free-living saprophytes in soils, fresh water and marine environments [42], were present at a low abundance (< 0.5%) in shrimp guts of both lake and river populations, but increased in lake-to-river shrimps (1.21%). Our sequence-based analysis indicated that the reads originated from the water environment, positively in agreement with the enrichment of this bacterial family in Kaoping River. This result suggests that lake shrimp might be subjected to a potential risk when moved to the river habitat. Another primary member in gut communities of lake-to-river shrimps, *Flavobacteriaceae*, proved to be acquired from multiple sources, including the host gut, river water, and sediment, according to our sequence-based analysis. Therefore, the origin of the pathogens could be species specific. On the other hand, a certain number of bacteria frequently identified as symbiotic microbes might impose positive effects on aquatic animals, e.g. *Thiotrichaceae* [43], *Cytophagaceae* [44], *Microbacteriaceae* [45, 46], as well as *Chitinophagaceae* and *Sphingomonadaceae* [47]. Several surveys identified members of *Thiotrichaceae* that appear to preferentially form ectosymbiotic relationships with marine invertebrates and likely have ecological and evolutionary significance in chemically challenging habitats. A recent study found that colonization by *Microbacterium* sp. can significantly improve shrimp larval survival and is considered to be a novel probiotic bacteria [48], yet it remains a challenge to investigate to what extent other environmental bacteria contribute to their host phenomic plasticity. Otherwise, in river-to-lake shrimp, we observed the family *Incertae Sedis XII* within the order *Bacillales*, among which all reads were classified to genus *Exiguobacterium*, which is also one of the putative probiotics in aquatic environments [49], and its sister genus *Bacillus* has been widely used as a probiotic for aquaculture [50]. These species of bacteria are commonly found in water and sediments and therefore are naturally ingested by shrimp that feed in or on the environmental material. Therefore, our findings provide the new perspective that the appropriate environment can promote the growth of commensal *Bacillales* in shrimp.

Global climate change has increased the incidence of rapid environmental change events, which might occur within the timescale of a single generation [51]. Thus, the surveys, which investigated the impact of rapid habitat change on gut microbiota, do not just provide the information on compositional change of gut microbes, but also represent the origin of the resulting reorganized community. The results of our sequence-based analysis provide the first evidence showing that both environmental and indigenous gut microbes contribute to the variation of gut microbiome composition, and might consequently increase the plasticity of the shrimp gut microbiome. Remarkably, when shrimp change habitat, proliferation of specific bacteria may originate from indigenous gut microbes or the environment. The explanation for the different preferred sources is not clear; nevertheless, the predominant effect of indigenous gut microbes rather than obtained the same species from the environment might be associated with the inheritance of gut microbes. Studies of other arthropod animals indicate that transmission pathways have fundamental influence on microbial symbiont persistence and evolution. For example, bees may acquire certain putative probiotics through maternal inheritance (vertical transmission) [52], while the core gut microbiome of honey bees is transmitted socially via hive surfaces [53], and the initial establishment of symbiotic gut bacteria in the turtle ant occurs after pupation via oral-rectal trophallaxis [54]. The shrimp usually care for the fertilized eggs, however the eggs are also exposed to environmental water with microbes, and the mechanism of selecting indigenous gut microbes might be the key to answer the inheritance of gut microbes in shrimp. Otherwise, we cannot rule out the priority effects [55–57] also contributed to this pattern. During community assembly, earlier colonists, such as indigenous gut microbes and even those acquired from the environment, may fill niche space from which subsequent microbes from the environment will be excluded, resulting in a predominant role of the indigenous bacteria in the microbiota plasticity. Accordingly, more experiments are required to evaluate how the shrimp transmit gut microbes and how microbes interact in assembly processes shaping microbial communities.

## Materials and methods

### Ethics statement

An ethics statement is not required for this survey. No specific permissions were required for the described field studies. The field location is not privately owned or protected in any way, and the field studies did not involve endangered or protected species.

### Field collections and habitat-exchange manipulation

To investigate the divergence of the shrimp gut microbiome and its association with distinct aqueous living environments, we harvested individual shrimp from each of two types of natural habitats: Chengcing Lake (“C”; reservoir; N 22° 39’ 57”, E 120° 21’ 2”; 8 shrimp) and Kaoping River estuary (“K”; N 22° 30’ 22”, E 120° 24’ 52”; 11 shrimp) in southern Taiwan (Fig 1). Simultaneously, environmental samples of water and sediment were collected from each of these habitats. Three lake shrimp (C) and six river shrimp (K) were selected as control groups, and the remaining wild-harvested shrimp (five from each habitat) were used for habitat-exchange experiment. Habitat-exchanged shrimp were cultured in the exchanged habitats for one month. All shrimp samples were immediately iced or frozen on dry ice after capture and stored at -20°C until workup. For each group, five shrimp of similar weights were collected for dissection. The microbial community was analyzed by the well-established pipeline described below and the comparison between communities was illustrated via statistical models.

The shrimp samples were aseptically washed with 70% EtOH and instruments were flame sterilized prior to dissection. Samples were kept in 1.75mL eppendorf tubes with 0.1mL distilled water and homogenized by 1.5mL disposable pestles (SSI-plastics, USA). The bacterial DNA from homogenized intestines and environments (water and sediment) were extracted using DNeazy Blood and Tissue kit and QIAamp DNA Stool Mini Kit (Qiagen, GmbH, Hilden, Germany), respectively, and quantified by Qubit 2.0 Fluorometer (Invitrogen, Life Technologies, CA., USA). Subsequent analysis was conducted with DNA mixtures containing equivalent amounts of DNA from the pooled samples. All procedures were performed in a laminar flow cabinet.

### Amplification, Illumina sequencing and analysis of gut microbial communities

The first two hypervariable regions (V1 and V2) of the small subunit ribosomal RNA gene were amplified using universal eubacterial primers. The forward primer 27F (5’-AGAGTTTGATCMTGGCTCAG-3’) and the reverse primer 355R (5’-GCTGCCTCCC- GAGGAGT-3’) [45, 58] were fused with Illumina overhang adapters and sample specific ten-nucleotide barcodes to allow multiple samples to be analyzed in parallel on a single picotiter plate. The pooled DNA was amplified with PCR (Taq DNA Polymerase 2x Master Mix Red, Biomol, GmbH, Germany) under the following running conditions: initial denaturation at 95°C for 5 min, 35 cycles of 1 min at 95°C, 45s at 55°C, 1 min at 72°C, and a final elongation step for 7 min at 72°C. All PCR products were confirmed using 2% agarose gel electrophoresis and subsequently isolated from the gel and purified by QIAquick gel extraction kit (Qiagen, Hilden, Germany). DNA concentrations of the cleaned PCR products were determined using the Quant-iT dsDNA HS assay kit and the Qubit fluorometer (Invitrogen, Carlsbad, CA, USA). The purified amplicons were further processed according to the Illumina standard protocol of 16S metagenomic sequencing library preparation, and sequenced by the MiSeq platform (Illumina, San Diego, Ca, USA) with the reagent kit v3. All datasets have been deposited in the Sequence Read Archive (SRA) database at NCBI under BioProject ID PRJNA354668.

The raw Illumina amplicon sequences were first demultiplexed, quality filtered, and analyzed using Mothur [59]. The criteria for filtering were read length (minimum of 200 and maximum of 600 bp), sequence quality score (minimum of 30), number of errors in the barcode (maximum of 1) and number of errors in the primer (maximum of 2). Barcode and primer sequences were removed from 5’ and 3’ends, and chimeras were checked and removed using the uchime_ref command in USEARCH [60]. After filtering and trimming processes, reads with an average length of 303 bp among all samples were used for downstream analyses. An UPARSE pipeline (usearch_global) [61] was used to cluster preprocessed reads into operational taxonomic units at 97% similarity. The bacterial 16S rRNA reference alignment sequence was exported from RDP [62], and OTUs were assigned into taxonomic hierarchy by Mothur (Classify.seqs) based on the reference sequences from RDP (version 9) [63]. To evaluate the fraction of species sequenced in each sample, rarefaction curves were generated by using fasta_rarify command in USEARCH. The microbial diversity was analyzed using Mothur based commands.

### Statistical analysis

A graphical environment for matrix visualization and cluster analyzer (GAP) [64] was used to generate hierarchical clustering and to present the abundance of grouped OTUs with a heatmap. Spearman’s rank was used to generate a correlation matrix among samples and grouped OTUs. Average-linkage was then used to calculate hierarchical clustering among samples based on the correlation matrix. In order to reduce noise within the data, only OTUs that made up more than 1% of the sequences of the library and had a more than 2-fold change after habitat-exchange manipulation were represented in the Figures, the rest of the OTUs were grouped as “others”. To perform a parallel comparison, we conducted the Non-metric multidimensional scaling (NMDS) ordination approach [65] based on OTUs abundance classified at the family level with R package vegan, gplots, and RcolorBrewer [66–68]. To reveal the shared OTUs among shrimp samples, Venn diagrams were produced using Venny 2.1.0 online freeware [69].

In order to investigate the roles of environmental specific bacteria (family level) in shrimp gut when exposed to different types of habitat, the compositional changes in gut microbial community were analyzed. The log2 fold change estimate was used to quantify the changes of OTU abundance (family-level) between native and habitat-exchanged shrimps and select the candidate targeted OTUs, which had significantly greater abundance and may play a critical role in gut of shrimp living in new habitat environment. The targeted OTUs with fold change ≥ 2.00 (|log2 ratio| ≥ 1) were regarded as biologically meaningful and selected for later analysis. Furthermore, to further determine the origin (from new habitat or original gut) of these sequences from targeted OTUs, a sequence-based analysis was performed. The reads from targeted OTUs of replicated samples were pooled and assigned to new operational taxonomic units with 99% similarity using the USEARCH global alignment algorithm [61]. These targeted OTUs were identified as ‘core’ OTUs if they appeared in ≥ 50% of replicate samples. Then, the origins of the sequences in the targeted OTUs were judged according to the UPARSE clustering manner with each microbial community (e.g. gut, water, sediment). The sequences of targeted OTUs in habitat-exchanged shrimp were measured and divided into G (sequences clustered with the gut microbiota of shrimp from the native habitat), S (sequences clustered with sediment microbiota of the exchanged habitat), W (sequences clustered with water microbiota of the exchanged habitat), SW (sequences clustered with sediment and water microbiota of the exchanged habitat), GS (sequences clustered with the gut microbiota of shrimp from the native habitat and sediment microbiota of the exchanged habitat), GW (sequences clustered with the gut microbiota of shrimp from the native habitat and water microbiota of the exchanged habitat), GSW (sequences clustered with the gut microbiota of shrimp from the native habitat, sediment, and water microbiota of the exchanged habitat), and Specific (sequences not clustered with any group). Note that reads that only appeared in one of the replicated shrimps were excluded from the analysis.

## Supporting information

S1 Table The diversity of bacterial communities in shrimp gut, water, and sediment. (DOCX)

S2 Table The environmental conditions of water area in Kaiping River and Chengcing Lake. (XLSX)

S1 Fig Bacterial community composition and enterotype groupings in relation to Phylum-level distribution. A heatmap was constructed using the heatmap.2 program within the gplots package for R.
(PDF)

S2 Fig Relationships between shrimp gut microbes and those from their habitats. In the network-based analysis of all gut microbes derived from shrimps and environments (a), the average relative abundance of OTUs in replicate samples was represented, and the Edge-weighted Spring Embedded algorithm as implemented in Cytoscape 3.4.0 was used to cluster the OTUs and hosts in this network. Sample nodes correspond to 97% OTUs (yellow circle: native shrimp; yellow triangle: habitat-exchanged shrimp; blue diamond: water; green square: sediment), while color of OTU nodes represent assignments of OTUs to samples nodes that are shared (yellow nodes: OTUs shared between two samples; orange OTU nodes: shared between 3 or 4 samples; pink OTU nodes: shared between 5 or 6 samples; red OTU nodes: shared between 7 or 8 samples). In the numbers of shared OTUs (b), the Venn diagrams were performed to reveal the number of OTUs shared among shrimp gut and environmental samples and produced in Venny 2.1.0 online freeware.
(PDF)

S3 Fig Alpha diversity levels (# OTUs) of environmental microbiota in Chengcing Lake and Kaoping River after rarefaction to 20313. Chao1 richness and Shannon diversity all varied between samples from lake and river (P < 0.001), except Chao1 between CS and KS (P = 0.0388).
(PDF)
