# Supplementary figures and images for: Habitat and indigenous gut microbes contribute to the plasticity of gut microbiome in oriental river prawn during rapid environmental change
Source: PLoS One. 2017 Jul 17;12(7):e0181427. doi: 10.1371/journal.pone.0181427 (PMC5513549; doi:10.1371/journal.pone.0181427)

**S1 Table. The diversity of bacterial communities in shrimp gut, water, and sediment.**
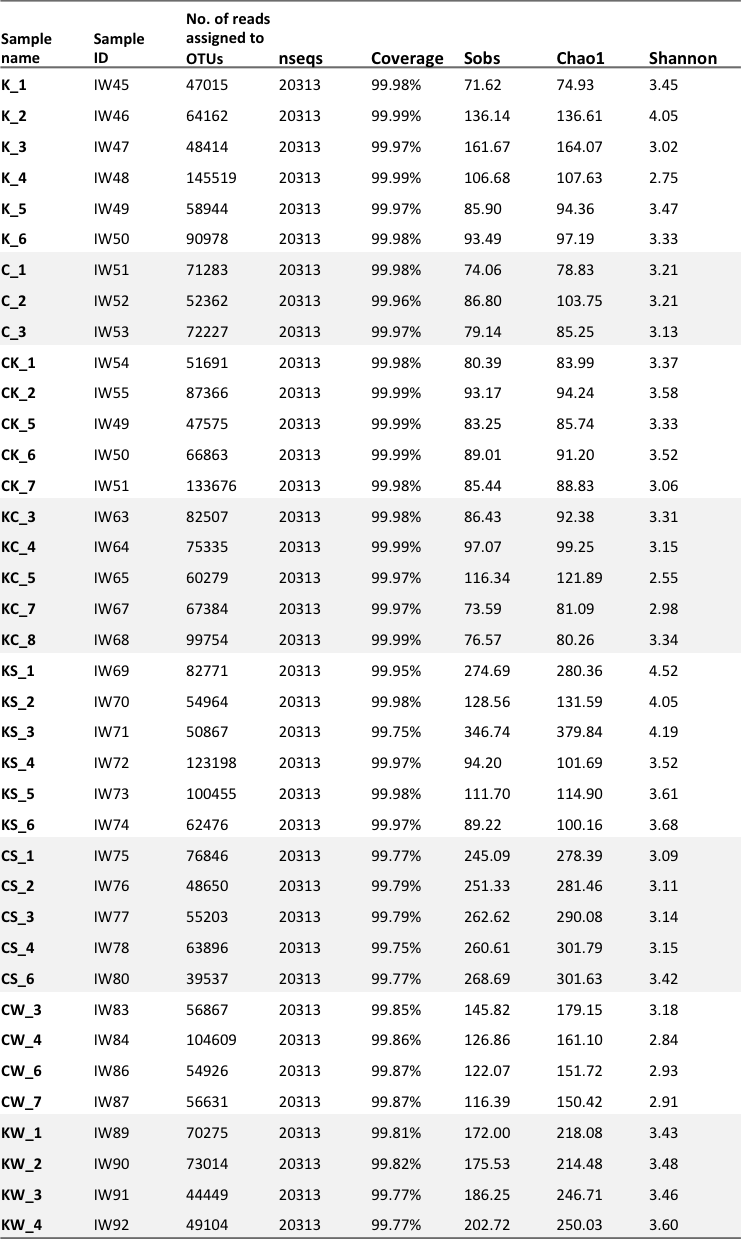

Supplement: S1 Table — (DOCX) [file pone.0181427.s001.docx]
