# Supplementary material for: Habitat and indigenous gut microbes contribute to the plasticity of gut microbiome in oriental river prawn during rapid environmental change
Source: PLoS One. 2017 Jul 17;12(7):e0181427. doi: 10.1371/journal.pone.0181427 (PMC5513549; doi:10.1371/journal.pone.0181427)
Supplement: S1 Fig — A heatmap was constructed using the heatmap.2 program within the gplots package for R. (PDF) [file pone.0181427.s003.pdf]

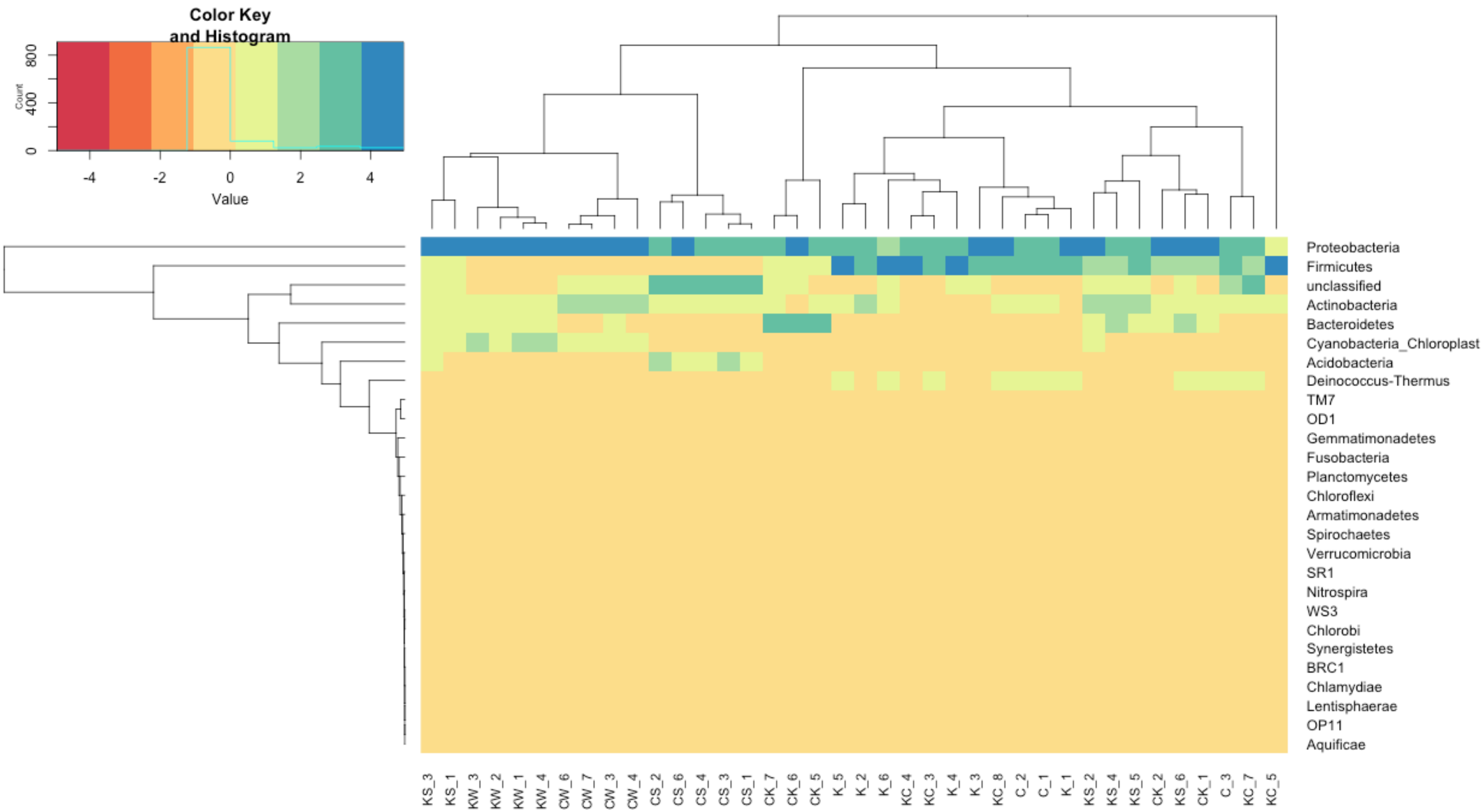

S1 Fig. Bacterial community composition and enterotype groupings in relation to Phylum-level distribution.

A heatmap was constructed using the heatmap.2 program within the gplots package for R.
