# Supplementary material for: Habitat and indigenous gut microbes contribute to the plasticity of gut microbiome in oriental river prawn during rapid environmental change
Source: PLoS One. 2017 Jul 17;12(7):e0181427. doi: 10.1371/journal.pone.0181427 (PMC5513549; doi:10.1371/journal.pone.0181427)
Supplement: S2 Fig — In the network-based analysis of all gut microbes derived from shrimps and environments (a), the average relative abundance of OTUs in replicate samples was represented, and the Edge-weighted Spring Embedded algorithm as implemented in Cytoscape 3.4.0 was used to cluster the OTUs and hosts in this network. Sample nodes correspond to 97% OTUs (yellow circle: native shrimp; yellow triangle: habitat-exchanged shrimp; blue diamond: water; green square: sediment), while color of OTU nodes represent assignments of OTUs to samples nodes that are shared (yellow nodes: OTUs shared between two samples; orange OTU nodes: shared between 3 or 4 samples; pink OTU nodes: shared between 5 or 6 samples; red OTU nodes: shared between 7 or 8 samples). In the numbers of shared OTUs (b), the Venn diagrams were performed to reveal the number of OTUs shared among shrimp gut and environmental samples and produced in Venny 2.1.0 online freeware. (PDF) [file pone.0181427.s004.pdf]

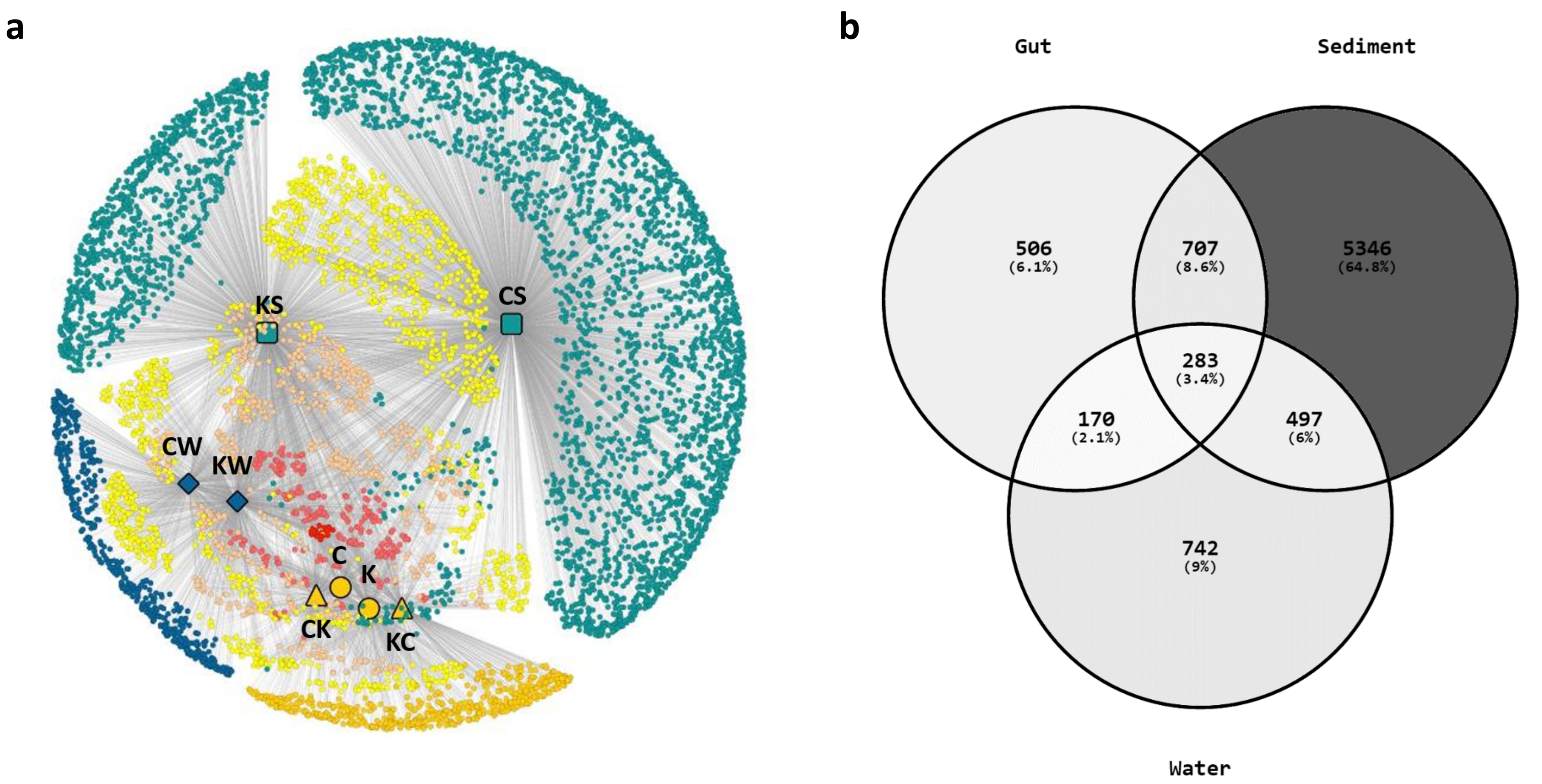

S2 Fig. Relationships between shrimp gut microbes and those from their habitats.
In the network-based analysis of all gut microbes derived from shrimps and environments (**a**), the average relative abundance of OTUs in replicate samples was represented, and the Edge-weighted Spring Embedded algorithm as implemented in Cytoscape 3.4.0 was used to cluster the OTUs and hosts in this network. Sample nodes correspond to 97% OTUs (yellow circle: native shrimp; yellow triangle: habitat-exchanged shrimp; blue diamond: water; green square: sediment), while color of OTU nodes represent assignments of OTUs to samples nodes that are shared (yellow nodes: OTUs shared between two samples; orange OTU nodes: shared between 3 or 4 samples; pink OTU nodes: shared between 5 or 6 samples; red OTU nodes: shared between 7 or 8 samples). In the numbers of shared OTUs (**b**), the Venn diagrams were produced in Venny 2.1.0 online freeware.
