# Supplementary material for: Habitat and indigenous gut microbes contribute to the plasticity of gut microbiome in oriental river prawn during rapid environmental change
Source: PLoS One. 2017 Jul 17;12(7):e0181427. doi: 10.1371/journal.pone.0181427 (PMC5513549; doi:10.1371/journal.pone.0181427)
Supplement: S3 Fig — Chao1 richness and Shannon diversity all varied between samples from lake and river (P < 0.001), except Chao1 between CS and KS (P = 0.0388). (PDF) [file pone.0181427.s005.pdf]

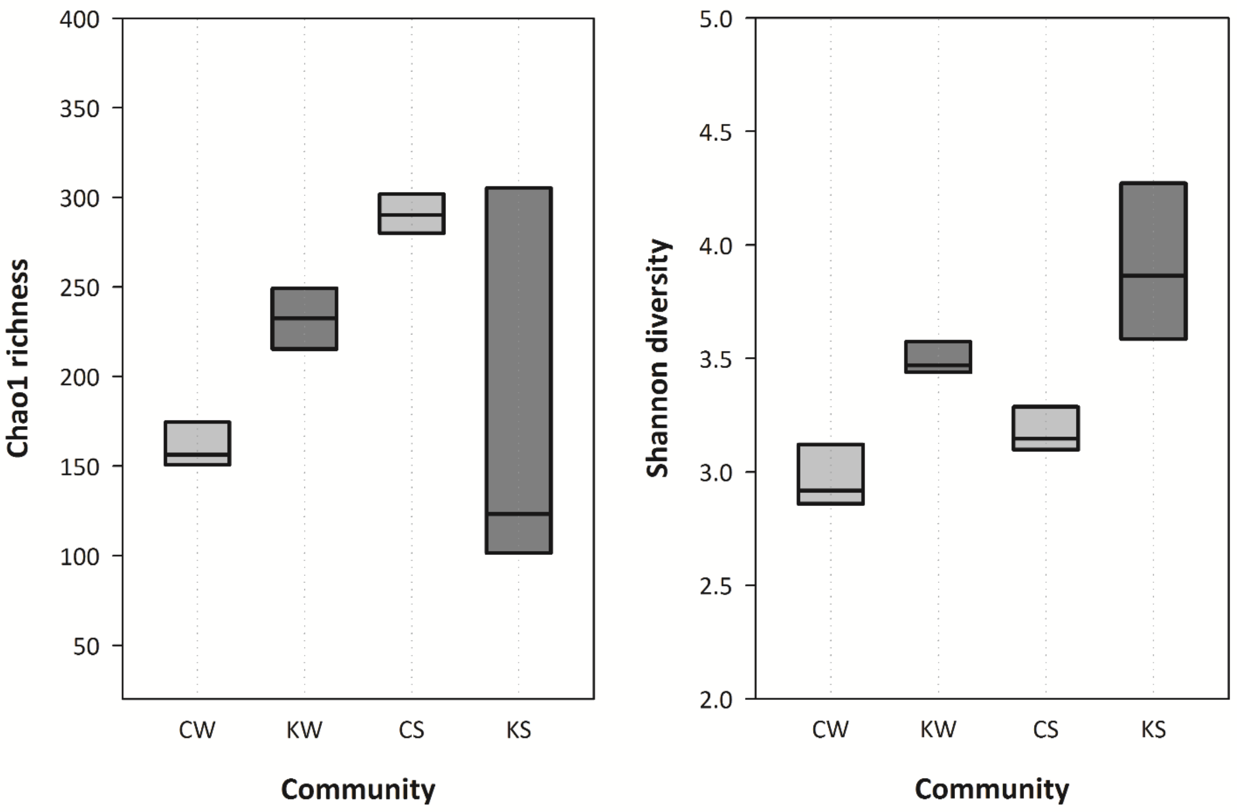

S3 Fig. Alpha diversity levels (# OTUs) of environmental microbiota in Chengcing Lake and Kaoping River after rarefaction to 20313. Chao1 richness and Shannon diversity all varied between samples from lake and river ($P < 0.001$), except Chao1 between CS and KS ($P = 0.0388$).
